# Analysis of right anterolateral impacts: the effect of head rotation on the cervical muscle whiplash response

**DOI:** 10.1186/1743-0003-2-11

**Published:** 2005-05-31

**Authors:** Shrawan Kumar, Robert Ferrari, Yogesh Narayan

**Affiliations:** 1 Physical Therapy, University of Alberta, 3–75 Corbett Hall, Edmonton, Alberta T6G 2G4, Canada; 2 Department of Medicine, University of Alberta, Edmonton, Alberta T6G 2B7, Canada; 3 Physical Therapy, University of Alberta, 3–78 Corbett Hall, Edmonton, Alberta T6G 2G4, Canada

**Keywords:** Cervical muscles, Electromyography, Acceleration, Anterolateral impacts, Whiplash

## Abstract

**Background:**

The cervical muscles are considered a potential site of whiplash injury, and there are many impact scenarios for whiplash injury. There is a need to understand the cervical muscle response under non-conventional whiplash impact scenarios, including variable head position and impact direction.

**Methods:**

Twenty healthy volunteers underwent right anterolateral impacts of 4.0, 7.6, 10.7, and 13.0 m/s^2 ^peak acceleration, each with the head rotated to the left, then the head rotated to the right in a random order of impact severities. Bilateral electromyograms of the sternocleidomastoids, trapezii, and splenii capitis following impact were measured.

**Results:**

At a peak acceleration of 13.0 m/s^2^, with the head rotated to the right, the right trapezius generated 61% of its maximal voluntary contraction electromyogram (MVC EMG), while all other muscles generated 31% or less of this variable (31% for the left trapezius, 13% for the right spleinus. capitis, and 16% for the left splenius capitis). The sternocleidomastoids muscles also tended to show an asymmetric EMG response, with the left sternocleidomastoid (the one responsible for head rotation to the right) generating a higher percentage (26%) of its MVC EMG than the left sternocleidomastoid (4%) (p < 0.05). When the head is rotated to the left, under these same conditions, the results are reversed even though the impact direction remains right anterolateral.

**Conclusion:**

The EMG response to a right anterolateral impact is highly dependent on the head position. The sternocleidomastoid responsible for the direction of head rotation and the trapezius ipsilateral to the direction of head rotation generate the most EMG activity.

## Background

Although many diagnostic efforts over the decades have aimed at objectively identifying the acute whiplash injury that is often labelled as "soft tissue injury" or "neck sprain", with the exception of a few case reports and excluding spinal cord or bony injury, the pathology of the acute whiplash injury remains elusive [1]. In the absence of an identifiable injury, efforts have simultaneously focused on development of better preventative measures and treatment approaches. Even without knowing what the acute whiplash injury is, for example, knowing more of the human response to whiplash type impacts led to the introduction of head restraints in 1969[2] and further innovations of head restraints have followed as the knowledge has increased [3]. Most efforts to understand the whiplash injury mechanism have focused on rear impacts [4-11]. Although it has been traditionally reported that rear-impacts account for most cases of whiplash injury, epidemiological evidence suggests that rear, lateral, and frontal collisions account for whiplash injury in roughly equal proportions [12].

Frontal collisions thus require more investigative attention, and yet there are a number of variables to consider in terms of understanding how the cervical muscles respond to a whiplash-type frontal impact. First, not all collision victims have their head in the neutral (facing forward) position. We recently reported on the effect of head rotation in straight-on frontal impacts [13], and compared this to the head in neutral position in a frontal impact [14]. With the head in neutral position, a frontal impact causes the greatest EMG activity to be generated symmetrically in the trapezii, which have an EMG activity that is 30–50% of their maximal voluntary contraction (MVC EMG). In a frontal impact with head rotated to the left, however, the left trapezius generated 77% of its maximal voluntary contraction (MVC) EMG (more than double the response of other muscles). In comparison, the right trapezius generated only 33% of its MVC. The right sternocleidomastoid (25%) and left splenius muscles (32%), the ones responsible for head rotation to the left, were more active than their counterparts. On the other hand, with the head rotated to the right, the right trapezius generated 71% of its MVC EMG, while the left trapezius generated only 30% of this value. Again, the left sternocleidomastoid (27% of its MVC EMG) and right splenius (28% of its MVC EMG), being responsible for head rotation to the right, were more active than their counterparts. Thus, head rotation produces an asymmetric EMG response.

Then there is the direction of impact. Frontal impacts are not always straight-on impacts. We have considered the example of a right anterolateral impact [15], and the results confirm the importance of direction of impact on the cervical muscle response. When the impact is a right anterolateral impact, the left trapezius still generated the greatest EMG, up to 83% of the maximal voluntary contraction EMG, and the left splenius capitis instead became more active and reached a level of 46% of this variable [15]. This is greater than the response of the splenius capitis in straight-on frontal impacts. Thus, direction of impact also determines which muscles respond and the proportionality of the response among the different muscle groups.

The question is whether head rotation in anterolateral impacts will increase or decrease the EMG activity, and how. We thus undertook a study to assess the cervical muscle response in right anterolateral impacts, but with the head rotated to either the left or right at the time of impact. This is part of a series of experiments to approach the more complex impact scenarios of varying directions and head positions.

## Materials and methods

### Sample

The methods for this study of offset frontal impacts are the same as that used previously for our previous right anterolateral and frontal impact studies [13-15]. Twenty healthy normal subjects (10 males, 10 females, all right-hand dominant) with no history of whiplash injury and no cervical spine pain during the preceding 12 months volunteered for the study. The study was approved by the University Research Ethics Board. The twenty subjects had a mean age of 23.6 ± 3.0 years, a mean height of 172 ± 7.7 cm, and a mean weight of 69 ± 13.9 kg.

### Tasks and Data Collection

**Figure 1:**
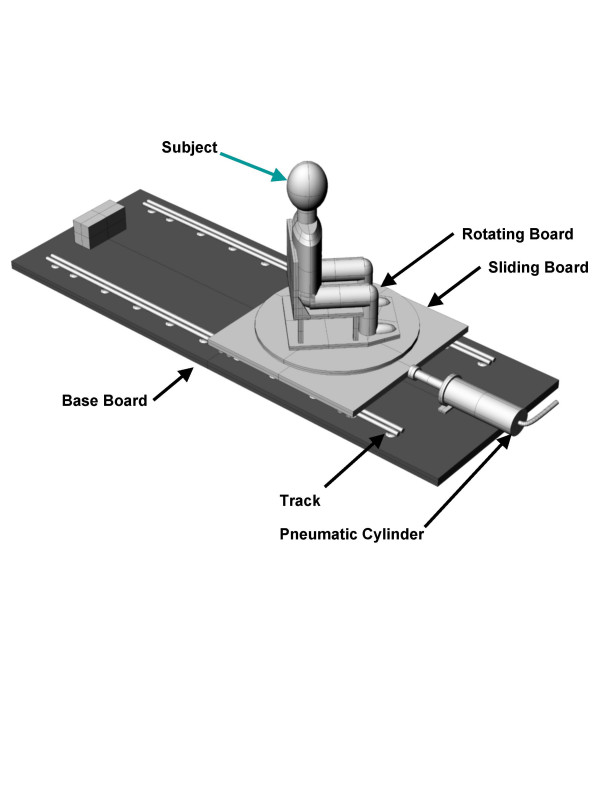
Illustration of the sled device for whiplash-type impacts.

Active surface electrodes with 10 times on-site amplification were placed on the belly of the sternocleidomastoids, upper trapezius at C4 level, and splenius capitis in the triangle between sternocleidomastoids and trapezii bilaterally. The fully-isolated amplifier had additional gain settings up to 10, 000 times with frequency response DC-5 kHz and common mode rejection ratio of 92 dB. Before calibrating sled acceleration, the cervical strength of the volunteers was measured to develop force-EMG calibration factor [16,17]. The seated and stabilized subjects exerted their maximum isometric effort in attempted flexion, extension, and lateral flexion to the left and the right for force-EMG calibration, as described by Kumar et al.[16,17]. The acceleration device consisted of an acceleration platform and a sled. The full details of the device and the electromyography data collection are given by Kumar et al.[7] and the device is as shown in Fig. 1. After the experiment was discussed and informed consent obtained, the age, weight, and height of each volunteer was recorded. The volunteers then were seated on the chair and stabilized in neutral spinal posture. The chair was rigid so as to minimize any effect of elastic properties of the chair following acceleration. Subjects were then outfitted with triaxial accelerometers (Model # CXL04M3, Crossbow technology, Inc., San Jose, California, U. S. A.) on their glabella and the first thoracic spinous process. Another triaxial accelerometer was mounted on the sled, not the chair. The accelerometers had a full scale nonlinearity of 0.2%, dynamic range of ± 5 g, with a sensitivity of 500 mV/g, resolution of 5 mg within a bandwidth of DC-100 Hz, and a silicon micromachined capacitive beam that was quite rugged and extremely small in die area. The subjects were then exposed to right anterolateral impacts (offset from a frontal impact by 45 degrees) with their head rotated 45 degrees to their left and right at accelerations of 4.0, 7.6, 10.7, and 13.0 m/s^2 ^generated in a random order by a pneumatic piston. To release the piston the solenoid of the pneumatic system was activated by an electronic impulse which was recorded for timing reference. Upon delivery of impact by the pneumatic piston, the sled moved on two parallel tracks mounted 60 cm. apart. The coefficient of friction of the tracks was 0.03 which allowed for smooth gliding of the sled on the rails. The opposite end of the track was equipped with non-linear springs and high density rubber stopper to prevent the subject from sliding off the platform. Each subject effectively underwent 4 levels of accelerative impacts under two conditions of head rotation, for one direction of impact (a total of 8 impacts). The head rotation itself did not place the head in a more forward position. Although the subjects are asked to rotate their head prior to impact, nothing was done to fix the position, and the head is free to move after impact. The accelerations involved in this experiment were low enough that injury was not expected. The acceleration was delivered in a way that mimicked the time course seen in motor vehicle collisions and occurred fast enough to produce eccentric muscle contractions. The acceleration impulse reached its peak value in 33 ms. Subjects were asked to report any headache or other aches they experienced in the days following the impacts.

### Data analysis

The data on the peak and average accelerations in all three axes of the sled, shoulder, and head for all four levels of accelerative impacts were measured. The gravity bias was eliminated by subtracting this value from the accelerometer readings. The onset of acceleration was measured by dropping the ascending slope line on the base line. The point of intersection of these lines was considered as onset of acceleration. In the analysis, the sample of volunteers was collapsed across gender because preliminary analysis showed no statistically significant differences in the EMG amplitudes between the men and women. The sled velocity and its acceleration subsequent to the pneumatic piston impact and the rubber stopper impact were measured. All timing data (time to onset of EMG and peak EMG) were referred to the solenoid of the piston firing. The time of the peak accelerations of sled and head were measured. Also, the time relations of the onset and peak of the EMG were measured and analyzed. The time to onset was determined when the EMG perturbation reached 2% of the peak EMG value to avoid false positives due to tonic activity. This method was chosen to avoid any false positives due to tonic EMG. This method was in agreement with projection of the line of slope on the baseline. EMG amplitudes were normalised against the subjects' maximal voluntary contraction electromyogram. The ratio percentage of the EMG amplitude versus the maximal contraction normalised EMG activity for that subject allowed us to determine the force equivalent generated due to the impact for each muscle.

Statistical analysis was performed using the SPSS statistical package (SPSS Inc., Chicago, IL) to calculate descriptive statistics, correlation analysis between EMG and head acceleration, analysis of variance (ANOVA) of the time to EMG onset, time to peak EMG, average EMG, and the force equivalents. Additionally, a linear regression analysis was carried out for the kinematic variables of head displacement, head velocity and head acceleration and EMG variables on the peak of the sled acceleration. Initially, all regressions were carried out to the level of exposure and subsequently they were extrapolated to twice the level of acceleration used in the study. The purpose of the regression analysis was to see if using the acceleration of the sled – one could predict the head acceleration and EMG response. The regression analysis was carried out using linear and non-linear functions. The linear regression was found to be the best fit, perhaps because the input acceleration impulse was non-linear.

## Results

### Head acceleration

**Figure 2:**
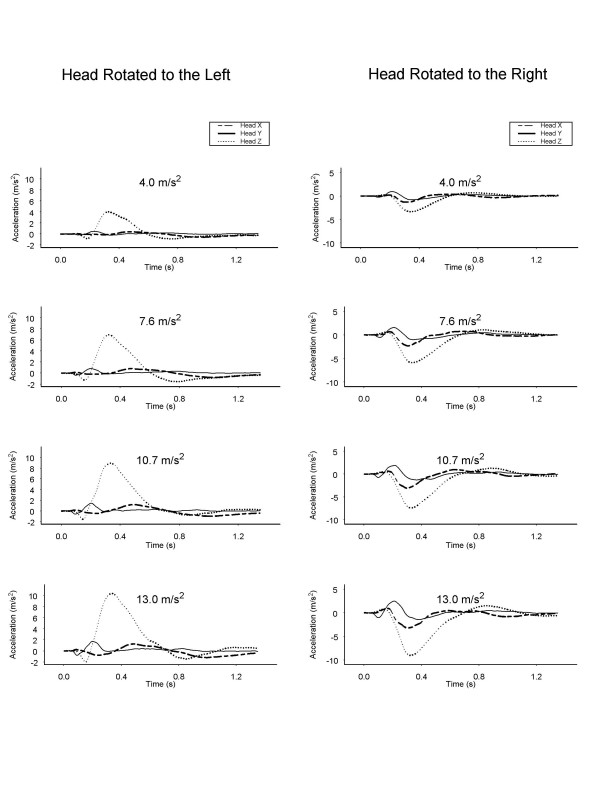
Head acceleration in the x, y, and z axes of one subject in response to the level of applied acceleration. The z-axis is parallel, the x-axis orthogonal, and the y-axis vertical to the direction of travel. Head X, head acceleration in the x-axis; Head Y, head acceleration in the y-axis; Head Z, head acceleration in the z-axis.

The kinematic response of the head to the four levels of applied acceleration are shown in Fig. 2. As anticipated, an increase in applied acceleration resulted in an increase in excursion of the head and accompanying accelerations (p < 0.05). The accelerations in these impacts were not associated with any reported symptoms in the volunteers.

### Electromyogram amplitude

In a right anterolateral impact, with the head rotated 45 degrees to the right or left, the trapezius muscle ipsilateral to the direction of head rotation showed the greatest EMG response (p < 0.05). The sternocleidomastoid muscles responsible for the head rotation each showed more EMG response to the pertubation than their counterparts (p < 0.05).

At a peak acceleration of 13.0 m/s^2^, for example with the head rotated to the right, the right trapezius generated 61% of its maximal voluntary contraction electromyogram, while all other muscles generated 31% or less of this variable. Though they generated less EMG activity, the sternocleidomastoids muscles also tended to show an asymmetric EMG response, with the left sternocleidomastoid (the one responsible for head rotation to the right) generating a higher percentage (26%) of its maximal voluntary contraction electromyogram than the right sternocleidomastoid (4%) (p < 0.05). When the head is rotated to the left, under these same conditions, the EMG results are reversed even though the impact direction remains right anterolateral. When looking left, the left trapezius generated 51% of its maximal voluntary contraction electromyogram, with only 14% of the maximal voluntary contraction for the right trapezius, and less than 25% for the remaining muscles. The sternocleidomastoid muscles in this case still showed an asymmetric EMG response, with the right sternocleidomastoid (the one responsible for head rotation to the left) generating a higher percentage (22%) of its maximal voluntary contraction electromyogram than the left sternocleidomastoid (4%) (p < 0.05).

**Figure 3:**
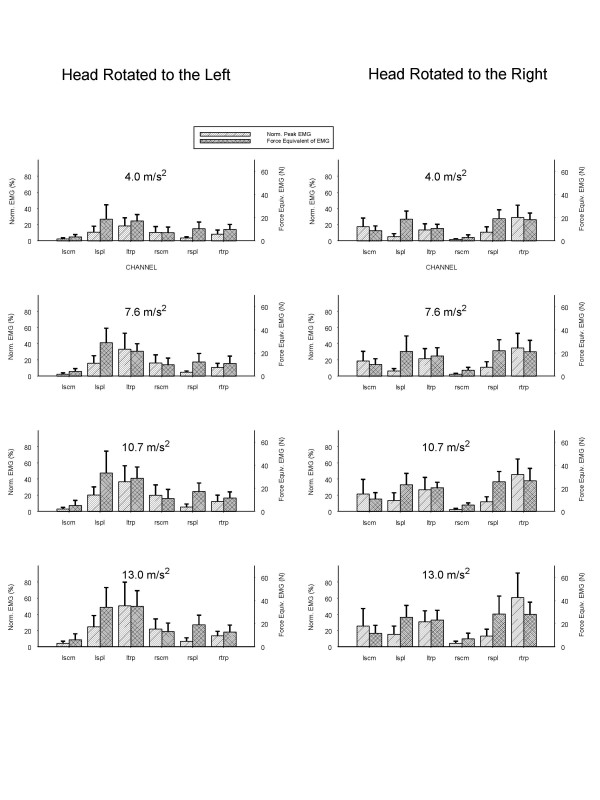
Normalized average and peak electromyogram (EMG) (percentage of isometric maximal voluntary contraction), force equivalent of EMG (N), and head rotated right or left, and applied acceleration. LSCM, left sternocleidomastoid; RSCM, right sternocleidomastoid; LSPL, left splenius capitis; RSPL, right splenius capitis; LTRP, left trapezius; RTRP, right trapezius.

**Figure 4:**
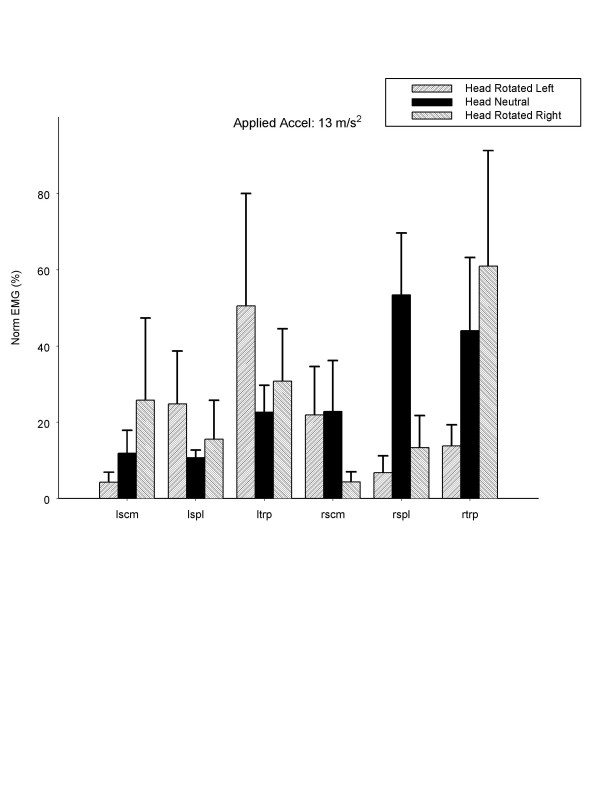
Normalized peak electromyogram (EMG) (percentage of isometric maximal voluntary contraction), for head in neutral position, rotated right, or rotated left, at an applied acceleration of 13.0 m/s^2^. LSCM, left sternocleidomastoid; RSCM, right sternocleidomastoid; LSPL, left splenius capitis; RSPL, right splenius capitis; LTRP, left trapezius; RTRP, right trapezius.

The normalized EMG for the sternocleidomastoid (SCM), splenius capitis (SPL) and trapezius (TRP) muscles are shown in Fig. 3. As the level of applied acceleration in the impact increased, the magnitude of the EMG recorded from the trapezius ipsilateral to the head rotation increased progressively and disproportionately compared to other muscles (p < 0.05). The reverse occurred when the head was rotated to the left, where the left TRP instead generated 77% of its MVC and again the remaining muscles generated 33% or less of their MVC. Figure 4 also compares these responses at the highest level of acceleration to the cervical muscle responses with the head in neutral position. The results indicate that head rotation affected the muscle response independent of direction of impact. Although the data concerning EMG responses with the head in neutral posture are from a different group of subjects, the methodology of always normalizing the EMG response to an individual's maximal voluntary contraction helps to adjust for these variables (i.e, gender, stature and age affects maximal voluntary contraction, and EMG responses should thus be normalized before making comparisons among individuals or groups). Thus, we were able to compare normalized populations from different studies, each group undergoing the same experimental protocols are used.

### Timing

The time to onset of the sled, shoulder, and head acceleration onset in the z-axis (axis along impact direction) and the EMG signals of the six muscles examined for head rotated to the left or right are presented in Table 1. The timing data is in relation to firing of the solenoid of the piston. The time to onset of the sled, torso, and head acceleration decreased with increased applied acceleration (p < 0.05). Similarly, the time to onset of the EMG show a trend (p > 0.05) for all muscles to decrease with increased applied acceleration. The mean times at which peak EMG occurred for all the experimental conditions are presented in Table 2, and also show a trend to earlier times of peak activity with increasing acceleration, though this again did not reach statistical significance.

**Table 1:** Mean Time to Onset (msec) of Acceleration and of Muscle EMG From the Firing of the Solenoid of the Pneumatic Piston

				Muscle					
	
				Sternocleidomastoid		Splenius Capitis		Trapezius	
	
Acceleration (m/s^2^)	Sled	Shoulder	Head	Left	Right	Left	Right	Left	Right
Right Head Rotation									
4.0	44 (19)	65 (32)	85 (17)	199 (116)	224 (136)	125 (45)	104 (52)	105 (44)	108 (48)
7.6	34 (10)	52 (18)	61 (21)	177 (81)	197 (143)	109 (33)	97 (40)	104 (42)	96 (46)
10.7	30 (11)	42 (14)	55 (21)	170 (49)	141 (109)	104 (42)	96 (47)	97 (33)	92 (41)
13.0	26 (11)	35 (15)	52 (21)	132 (60)	125 (63)	91 (22)	93 (36)	89 (30)	90 (28)
Left Head Rotation									
4.0	48 (21)	64 (26)	97 (22)	185 (61)	222 (50)	114 (43)	196 (105)	137 (35)	180 (55)
7.6	31 (15)	49 (22)	71 (25)	99 (45)	194 (45)	98 (37)	172 (78)	106 (45)	114 (44)
10.7	29 (14)	43 (12)	65 (22)	86 (47)	181 (77)	94 (35)	163 (107)	98 (41)	110 (48)
13.0	27 (11)	42 (19)	64 (19)	79 (48)	180 (70)	85 (27)	138 (48)	78 (29)	101 (36)

Times for the sled, shoulder, and head represent the time at which acceleration in z-axis (direction of travel) began. Times for the cervical muscles represent the time to onset for EMG activity. Values in parentheses represent one standard deviation.

**Table 2:** Mean Time (msec) at Which Peak Electromyogram Occurred After the Firing of the Solenoid of the Pneumatic Piston

	Muscle EMG					
	
	Sternocleidomastoid		Splenius Capitis		Trapezius	
			
Acceleration (m/s^2^)	Left	Right	Left	Right	Left	Right
Right Head Rotation						
4.0	479 (298)	599 (374)	247 (46)	264 (374)	223 (20)	228 (28)
7.6	379 (281)	569 (263)	225 (36)	224 (32)	211 (28)	227 (24)
10.7	363 (212)	547 (414)	219 (36)	219 (30)	206 (31)	224 (35)
13.0	321 (225)	521 (349)	210 (35)	211 (23)	196 (30)	210 (26)
Left Head Rotation						
4.0	526 (342)	687 (433)	243 (34)	822 (511)	281 (90)	664 (255)
7.6	255 (72)	576 (141)	227 (19)	704 (365)	267 (42)	262 (60)
10.7	245 (34)	521 (240)	223 (27)	631 (225)	256 (57)	246 (58)
13.0	244 (25)	510 (284)	215 (32)	608 (208)	249 (52)	218 (55)

Values in parentheses represent one standard deviation.

The relationship between the force equivalent EMG response of each muscle and the head acceleration are shown in Table 3. To obtain the force equivalency of a muscle response due to impact, we first performed a linear regression analysis on the graded EMG data obtained in the maximal voluntary contraction trials. This resulted inan equation for force/emg ratio. EMG values from each muscle as measured in this impact study were then entered into the equation, giving us a force equivalent value (Newtons) for each muscle as shown in Table 3. The kinematic responses show that very-low velocity impacts produce less force equivalent than the maximal voluntary contraction for the same subject. The head accelerations were correspondingly lower than the sled accelerations in this experiment. For very-low velocity impacts, this is to be expected, as it is usually only when the sled acceleration exceeds 5 g's that head acceleration begins to exceed sled acceleration. This experiment involved less than 2 g accelerations.

**Table 3:** Mean Force Equivalents (Newtons, N) and Mean Head Accelerations at Time of Maximal EMG in Direction of Travel for Right Anterolateral Impact.

	Force Equivalents for Muscle (N)						
		
		Sternocleidomastoid		Splenius Capitis		Trapezius	
				
Sled Acceleration (m/s^2^)	Head Acceleration (m/s^2^)	Left	Right	Left	Right	Left	Right
Right Head Rotation							
4.0	3.6 (0.8)	9 (4)	3 (2)	19 (7)	19 (8)	11 (4)	18 (6)
7.6	6.1 (1.0)	10 (5)	5 (2)	21 (14)	22 (10)	18 (7)	21 (10)
10.7	8.0 (1.1)	11 (6)	6 (2)	23 (10)	26 (9)	21 (5)	27 (11)
13.0	9.7 (1.4)	12 (7)	7 (5)	26 (10)	18 (16)	23 (9)	28 (11)
Left Head Rotation							
4.0	4.3 (0.7)	4 (2)	7 (5)	19 (13)	11 (6)	17 (6)	10 (4)
7.6	7.7 (1.3)	4 (3)	10 (6)	29 (13)	12 (8)	22 (7)	11 (6)
10.7	10.0 (1.3)	5 (4)	11 (8)	33 (19)	17 (7)	29 (10)	12 (6)
13.0	11.7 (1.8)	6 (5)	13 (7)	34 (17)	19 (8)	35 (14)	13 (6)

Values in parentheses represent one standard deviation.

### Regression analyses

**Figure 5:**
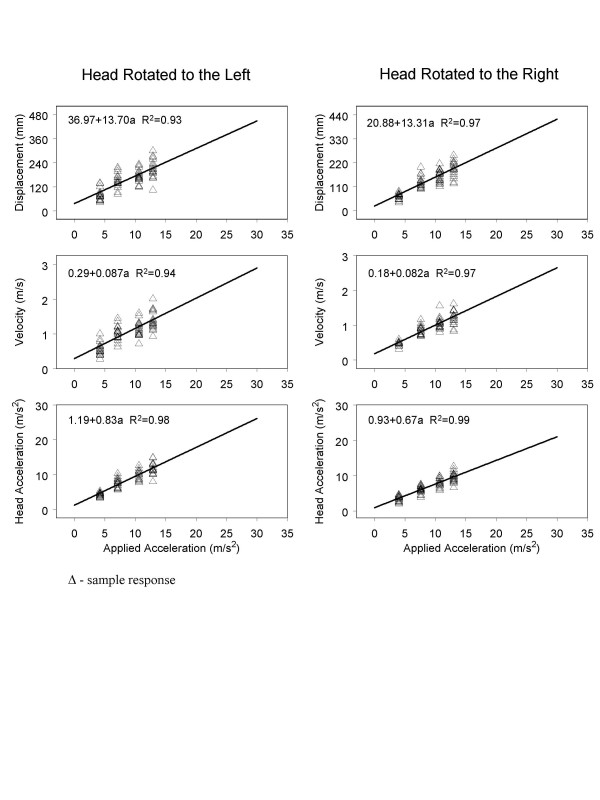
Extrapolated regression plots of the effect that applied acceleration has on the head motion variables of displacement (**A**) (mm), velocity (**B**) (m/s), and acceleration (**C**) obtained (m/s^2^).

The applied acceleration, and the muscles examined had significant main effects on the peak EMG activity (p < 0.05) as shown in Table 4. We used a linear regression model to plot the available data and extrapolate from the experimental accelerations to accelerations on the order of 30 m/s^2^. Initially, regression analyses were performed only up to 13.0 m/s^2 ^using a linear function. The kinematic variables of head displacement, velocity, and acceleration in response to applied acceleration were calculated (see Fig. 5.). Additionally, we also regressed the EMG magnitudes on acceleration. The responses of the left and right muscle groups were extrapolated to more than twice the applied acceleration value.

**Table 4:** ANOVA table for Peak EMG (μV) by Muscles and Applied Acceleration.

	Source	df	F	Sig.
Right Head Rotation	applied acceleration	3	13.38732	0.00
	muscle	5	64.17247	0.00
				
Left Head Rotation	applied acceleration	3	18.76792	0.00
	muscle	5	87.74690	0.00

## Discussion

The chief purpose of this study was to see what effect head rotation had on muscle responses in a right anterolateral impact. When the head was in neutral position in a previous study of right anterolateral impact [15], the left trapezius generated the greatest EMG, up to 83% of the maximal voluntary contraction EMG, and the left splenius capitis instead became more active and reached a level of 46% of this variable. In the current study, having kept the impact direction constant, but varying head rotation to right or left we see that the muscles responsible for head rotation (the contralateral sternocleidomastoid), and those which are likely stretched by this rotation (the ipsilateral trapezius), are most active and differ from their counterparts.

Although one might predict this, the human response to impacts and the neck structure is seemingly complex enough that it cannot always be assumed to be as one predicts. Our study methodology allowed for direct testing of the response rather than assumptions. There is no direct way to measure forces exerted by muscles due to neck perturbation and subsequent muscle activity, examining the EMG activity generated allows one to compare this to EMG activity in voluntary contractions. This in turn allows one to relate the muscle responses to normal muscle forces in various physiological ranges of activity. Because one cannot test the higher accelerations for ethical reasons, the best one can do currently is to compare to the small volunteer studies that were done previously. Further studies with larger samples and perhaps somewhat higher accelerations (within ethical limits) will allow to determine further how reasonable these extrapolations are. The projected values are hypothetical and likely to be affected by the ligaments and joint geometry in a manner different from that recorded in the experiment.

In frontal impacts, the direction of impact, anterolateral or straight-on, determines the muscle response, but so too does the occupant's head position, rotated right or left, at the time of impact. Anecdotally at least, whiplash patients report both offset impacts and also may report head rotation to the left or right at the time of impact. These patients also tend to emphasize the unilateral nature of their neck pain, but it remains to be seen in epidemiological studies if this is true. The evidence from low-velocity impacts studies does point in the direction of differential injury risks to different muscles depending on the impact conditions. This is in keeping with other studies of the pattern of muscle activation. Gabriel et al.[19] assessed maximal static strength and bilateral EMG activity associated with force exerted in the direction of the anatomic reference planes, as well as for planes at 30° intervals between the anatomic reference planes. In extending previous work in this area [19,20], Gabriel et al. observed that right-hand dominant subjects have the greatest strength directed to the right side of the body. For this reason, it is important to normalize EMG responses to impact to the subject's maximal voluntary contraction EMG, to account for directional and other confounders. Also, they showed that the SCM muscles are an agonist for static contractions with force exerted in a direction that corresponded to flexion, and a synergist for a force direction associated with lateral bending. It is thus expected that an anterolateral impact will generate the greatest response from the SCMs, and this is consistent with our findings.

Whether or not the pathology of the acute whiplash injury is known, measures to prevent this injury or understand its nature may well be advanced by understanding both the cervical muscle responses and the head kinematics in response to whiplash-type impacts. The difficulty is that besides individual subject characteristics, there are many collision parameters which may affect the pattern of response, including severity of impact, direction of impact, awareness of impending impact, head position, seat design and restraint systems. We have, however, begun the process of a larger series of investigations by showing what effect increasing acceleration, impact direction, head rotation and expectation has on muscle responses when other factors are held constant (i.e. seat and restraint type) [7,13-15]. Future studies can build on this and determine how different seat design or other factors that exist in vehicles affect muscle responses when things such as acceleration, expectation, and direction, for example, are held constant. EMG studies also allow one to examine muscle group responses and patterns, rather than simply describe head or other body region accelerations. The experimental design we have used to study neck perturbations to very low-velocity change is not intended to mimic vehicle occupancy, but rather to allow for the initial exploration of the role of EMG in assessing neck perturbations.

## Abbreviations

MVC (Maximal Voluntary Contraction); EMG (Electromyogram); cm (Centrimetres); dB (decibels); C4 (fourth cervical vertebra); mV/g (Millivolts per gram); Hz (Hertz); kHz (kilohertz); g (acceleration due to gravity); m/s2 (metres per second per second); kg (kilograms); SCM (Sternocleidomstoid); TRP (Trapezius); SPL (Splenius capitis)

## Competing Interests

The author(s) declare that they have no competing interests.

## Authors' Contributions

SK made substantial contributions to conception and design, to acquisition of data, and analysis and interpretation of data, was involved in drafting the article and revising it critically for important intellectual content. RF made substantial contributions to analysis and interpretation of data, and was involved in drafting the article and revising it critically for important intellectual content. YN made substantial contributions to acquisition of data, and analysis and interpretation of data. All authors read and approved the final manuscript.

## References

[B1] Ferrari R (1999). The Whiplash Encyclopedia The Facts and Myths of Whiplash.

[B2] Ruedmann AD (1969). Automobile safety device – headrest to prevent whiplash injury. JAMA.

[B3] Jakobsson L, Lundell B, Norin H, Isaksson-Hellman I (2000). WHIPS – Volvo's whiplash protection study. Accid Anal Prev.

[B4] Brault JR, Wheeler JB, Siegmund GP, Brault EJ, Weber M, Peuker C, Wortler K. (1998). Clinical response of human subjects to rear-end automobile collisions. Arch Phys Med Rehabil.

[B5] Castro WH, Schilgen M, Meyer S, Weber M, Peuker C, Wortler K (1997). Do "whiplash injuries" occur in low-speed rear impacts ?. Euro Spine J.

[B6] Kumar S, Narayan Y, Amell T (2000). Role of awareness in head-neck acceleration in low velocity rear-end impacts. Accid Anal Prev.

[B7] Kumar S, Narayan Y, Amell T (2002). An electromyographic study of low-velocity rear-end impacts. Spine.

[B8] Magnusson ML, Pope MH, Hasselquist L, Bolte KM, Ross M, Goel VK, Lee JS, Spratt K, Clark CR, Wilder DG (1998). Cervical electromyographic activity during low-speed rear-end impact. Euro Spine J.

[B9] Siegmund GP, Sanderson DJ, Myers BS, Inglis JT (2003). Awareness affects the response of human subjects exposed to a single whiplash-like perturbation. Spine.

[B10] Szabo TJ, Welcher J, Anderson RD (1994). Human occupant kinematic response to low speed rear-end impacts. Proceedings of the Thirty Eighth Stapp Car Crash Conference. Warrendale, Pennsylvania: Society of Automotive Engineers.

[B11] Szabo TJ, Welcher J (1992). Dynamics of low speed crash tests with energy absorbing bumpers. Warrendale, Pennsylvania: Society of Automotive Engineers.

[B12] Cassidy JD, Carroll LJ, Cote P, Lemstra M, Berglund A, Nygren A (2000). Effect of eliminating compensation for pain and suffering on the outcome of insurance claims for whiplash injury. N Engl J Med.

[B13] Kumar S, Ferrari R, Narayan Y Turning away from whiplash. An EMG study of head rotation in whiplash impact. J Orthop Res.

[B14] Kumar S, Narayan Y, Amell T (2003). Analysis of low-velocity frontal impacts. Clin Biomech.

[B15] Kumar S, Ferrari R, Narayan Y (2004). Cervical muscle response to whiplash-type right anterolateral impacts. Euro Spine J.

[B16] Kumar S, Narayan Y, Amell T (2001). Cervical strength of young adults in sagittal, coronal, and intermediate planes. Clin Biomech.

[B17] Kumar S, Narayan Y, Amell T, Ferrari R (2002). Electromyography of superficial cervical muscles with exertions in sagittal, coronal, and oblique planes. Euro Spine J.

[B18] Kumar S, Narayan Y, Amell T (2002). An electromyographic study of low-velocity rear-end impacts. Spine.

[B19] Spitzer WO, Skovron ML, Salmi LR, Cassidy JD, Duranceau J, Suissa S, Zeiss E (1995). Scientific monograph of the Quebec Task Force on Whiplash-Associated Disorders. Spine.

[B20] Gabriel DA, Matsumoto JY, Davis DH, Currier BL, An KN (2004). Multidirectional neck strength and electromyographic activity for normal controls. Clin Biomech.

[B21] Vasavada AN, Peterson BW, Delp SL (2002). Three-dimensional spatial tuning of neck muscle activation in humans. Exp Brain Res.

